# FGFR4 p.Gly388Arg polymorphism in PBMCs of LAM patients: findings of a pilot study

**DOI:** 10.3389/fmed.2025.1544910

**Published:** 2025-07-24

**Authors:** Sinem Koc-Gunel, Amy L. Ryan, Melanie Winter, Thomas O. F. Wagner

**Affiliations:** ^1^Department of Internal Medicine, Infectious Diseases, University Hospital Frankfurt, Goethe University Frankfurt, Frankfurt, Germany; ^2^Institute for Medical Virology, University Hospital Frankfurt, Goethe University Frankfurt, Frankfurt am Main, Germany; ^3^Department of Anatomy and Cell Biology, Carver College of Medicine, University of Iowa, Iowa City, IA, United States; ^4^Dr. Senckenberg Institute of Pathology, University Hospital Frankfurt, Frankfurt am Main, Germany; ^5^Frankfurt Reference Center for Rare Diseases (FRZSE), University Hospital Frankfurt, Goethe University Frankfurt, Frankfurt am Main, Germany; ^6^European Reference Network for Respiratory Diseases (ERN-Lung), University Hospital Frankfurt, Goethe University Frankfurt, Frankfurt am Main, Germany

**Keywords:** lymphangioleiomyomatosis (LAM), FGFR4 p.Gly388Arg polymorphism, peripheral blood mononuclear cells (PBMCs), forced expiratory volume (FEV₁%), next-generation sequencing (NGS), mTOR signaling pathway, genetic modifiers in rare lung disease, spatial transcriptomics

## Abstract

Lymphangioleiomyomatosis (LAM) is a rare, progressive lung disease characterized by neoplastic-like proliferation of abnormal smooth muscle–like cells, primarily driven by mutations in the *TSC2* gene. These mutations result in hyperactivation of the mTOR signaling pathway, leading to uncontrolled cell growth. However, additional genetic variants may modulate disease progression and severity. In this observational pilot study, we investigated the potential role of co-occurring variants. Peripheral blood mononuclear cells from seven sporadic LAM patients were analyzed using next-generation sequencing to identify potentially tumorigenic variants. The FGFR4 p.Gly388Arg gain-of-function polymorphism was identified in four patients, with allelic frequencies ranging from 49 to 99%. Patients with the variant exhibited significantly faster rates of lung function decline, as measured by FEV₁%, compared to those without the variant. Spatial transcriptomic analysis of LAM lung tissue revealed *FGFR4* expression predominantly in alveolar fibroblasts and AT2 epithelial cells, key compartments in lung remodeling, while detection in PBMCs supports a potential systemic role. These preliminary findings support the hypothesis that FGFR4 mutations contribute to the systemic aspects of LAM, potentially exacerbating disease severity. They also highlight the need for larger, mechanistic studies to evaluate FGFR4 as a biomarker or therapeutic target. Overall, this study provides a hypothesis-generating framework for future investigations into the genetic drivers of LAM beyond *TSC2* mutations.

## Introduction

Lymphangioleiomyomatosis (LAM) is a rare, progressive multisystem disorder characterized by the abnormal proliferation and phenotypic transformation of smooth muscle-like cells surrounding blood vessels, bronchioles, and lymphatic vessels (1). This pathological process leads to cystic lung lesions, progressive respiratory failure, and the development of tumors in the kidneys and retroperitoneum (2–5). While traditionally considered a benign lung disease, the World Health Organization classifies LAM as a perivascular epithelioid tumour (PeComa) due to its neoplastic features (6). LAM cells have been detected in a variety of body fluids, including the lymphatic system, and the disease has recurred in patients after lung transplantation, suggesting its metastatic potential (7). The mechanism by which LAM cells spread across organs or enter the circulatory system remains unclear. Despite their relatively low proliferative index, LAM cells exhibit several cancer-like characteristics. Recent studies, including our own, have identified activated αSMA-positive fibroblasts in LAM lesions, which exhibit increased invasiveness compared to fibroblasts from lung tissues with no evidence of chronic disease (8). Moreover, lymphangiogenesis and extracellular matrix (ECM) remodeling in LAM, driven by growth factors like VEGF-D, VEGF-A, and FGF7, closely mirror mechanisms observed in malignant cancers (9). The current understanding of LAM pathogenesis implicates somatic mutations in the *TSC2* gene in sporadic LAM, and germline mutations in *TSC1* or *TSC2* in cases associated with tuberous sclerosis complex. These mutations are often accompanied by loss of heterozygosity (LOH), particularly in *TSC2*, leading to constitutive activation of the mTOR signaling resulting in unchecked proliferation of smooth muscle-like cells and cytoskeletal dysregulation (10). Mutations in both genes vary widely, with no specific hotspots, including missense, large deletions, and in-frame deletions (11, 12). Treatment therefore currently focuses on targeting LAM effector cells with TSC mutations using rapamycin, an mTOR inhibitor (1). Unfortunately, rapamycin does not reverse existing lung damage or completely halt disease progression, underscoring the need for alternative or adjunctive therapeutic strategies. Neoplastic diseases often involve both driver mutations, directly promoting tumor growth, and co-driver mutations, influencing disease progression. While mutations *TSC2* are recognized as the primary driver, activating the mTOR pathway, in LAM additional mutations may contribute to disease progression. In this pilot study, we investigated the presence of additional genetic variants, specifically potential co-driver polymorphisms, in LAM and evaluated their potential clinical relevance within a hypothesis-generating framework.

## Methods

### Patient cohort

Seven sporadic-LAM patients were monitored at a large academic hospital in Germany over 8 years. Peripheral blood (5 mL) was collected from each patient via venipuncture using EDTA tubes. Clinical data, including lung function measurements (FEV1%), patient age, and treatment regimens were collected. Written informed consent was obtained from all patients and the study was approved by the Institutional Review Board (IRB) and the Ethics Committee (STO-02-2017). LAM was diagnosed based on standard diagnostic criteria according to American Thoracic Society (ATS) guidelines, which include typical cystic lung lesions identified on high-resolution CT (HRCT) scans, and in some cases through additional screening of serum VEGF-D levels (above 800 pg./mL) as a biomarker for LAM. In cases where VEGF-D testing was not conducted, diagnosis was confirmed by a combination of clinical and radiological findings. For five out of seven patients, the diagnosis was additionally confirmed through lung biopsy in conjunction with the radiological findings. Pulmonary function tests (PFTs) were performed at variable intervals, depending on clinical need, over follow-up periods ranging from 2 to 8 years, reflecting real-world observational data.

### PBMC isolation

PBMCs were isolated from whole blood with a standartized protocol using a Ficoll gradient. Briefly, whole blood was diluted 1:1 with phosphate-buffered saline (PBS) and gently layered over Ficoll-Paque in a sterile conical tube. Samples were centrifuged at 1200 × *g* for 20 minutes at room temperature without brake. The peripheral blood mononuclear cell (PBMC) layer, visible as a distinct cloudy ring at the plasma–Ficoll interface, was carefully collected, transferred to a new tube, and washed twice with PBS by centrifugation at 300 × *g* for 10 minutes to remove residual platelets and Ficoll.

### DNA extraction and quantification

Genomic DNA was (13) extracted using the Maxwell^®^ RSC Instrument (Promega Corporation, Madison, Wisconsin, United States) with the Maxwell^®^ RSC FFPE Plus DNA Kit (Promega Corporation), following the manufacturer’s protocol. DNA quantification was performed using the Qubit 4 Fluorometer (Invitrogen, Thermo Fisher, Waltham, Massachusetts, United States).

### Library preparation and sequencing

DNA libraries were prepared using 20 ng of input DNA and the Ion AmpliSeq^™^ Library Kit 2.0, following manufacturer’s instructions. The quality of the library preparation was evaluated using the 4,150 Tapestation System (Agilent Technologies, Waldbronn, Germany). Targeted variant analysis was performed using the Oncomine Comprehensive Assay v3 (Thermo Fisher Scientific), an NGS platform designed to detect single nucleotide variants (SNVs), insertions and deletions (INDELs), copy number variations (CNVs), and gene fusions across a wide panel of cancer-related genes. The libraries were pooled and subsequently sequenced on the Ion S5 System (Thermo Fisher Scientific). Raw sequencing data were processed using Torrent Suite™ software for primary analysis. Data analysis was performed using Ion Reporter™ Software (version 5.12.0.0) with the Oncomine™ Variants 5.10 and Oncomine™ Extended 5.12 filter chains. Quality control metrics for the sequencing data adhered to the platform’s cut-off values. Specifically, samples were required to meet stringent thresholds, including amplicon coverage >500x, at least 90% gene amplicon coverage (i.e., coverage across the amplified target regions) and 90% BED region coverage (genomic intervals defined by Browser Extensible Data files). Additionally, variant quality scores were assessed using a Phred score of ≥33 and Q ≥ 30 to ensure high-confidence variant calling. All variant calls, including those for FGFR4, were further validated through orthogonal visual inspection using the Integrative Genomics Viewer (IGV).

### Bioinformatics analysis

Post-sequencing data analysis focused on identifying relevant variants in the *FGFR4* gene, particularly the p.Gly388Arg polymorphism. Other tumorigenic mutations in genes such as TSC2, BRCA2, and NOTCH3 were also evaluated. The results were analyzed using Ion Reporter™, with identified variants cross-referenced against public databases for clinical relevance. The allelic frequency of each variant was calculated, and data were validated by cross-referencing with control samples, including the Seraseq^®^ Solid Tumor FFPE (Formalin-Fixed, Paraffin-Embedded) DNA Reference Material (SeraCare).

### Lung function assessment

Pulmonary Function Test (PFT), including spirometry and body plethysmography, were performed using a body plethysmograph following the recommendations of the ATS and the European Respiratory Society (ERS). The following parameters were measured: forced vital capacity (FVC), forced expiratory volume in 1 s (FEV1), FEV1/FVC ratio, residual volume (RV), residual volume/total lung capacity (RV/TLC), and diffusing capacity of the lungs for carbon monoxide (DLCO).

### Correlation analysis

To investigate the relationship between FGFR4 polymorphisms and lung function decline in LAM patients, two variables were analyzed: FGFR4 polymorphism status and FGFR4 allelic frequency. Polymorphism status was coded as 1 for the presence of the FGFR4 variant and 0 for its absence. FEV1% decline was used as a measure of lung function deterioration. To standardize longitudinal lung function measurements across patients, each pulmonary function test (PFT) was assigned a time value in “years from baseline”. The baseline was defined as the date of each patient’s earliest available PFT. Subsequent timepoints were calculated as the number of days elapsed from baseline, divided by 365.25 to account for leap years. Pearson’s correlation analysis was performed to assess the association between FGFR4 polymorphism status and FEV1% decline per year. A scatter plot was generated in R (version 2024.04.2 + 764) using the ggplot2 package, with FEV1% decline plotted on the y-axis and FGFR4 polymorphism status on the x-axis. A linear regression model was applied to visualize the relationship, and a fitted regression line was included in the plot. The association between FEV1% decline and FGFR4 allelic frequency was also analyzed using Pearson’s correlation. For this analysis, allelic frequency was treated as a continuous variable, and the ggplot2 package was used to create scatter plots and regression lines to visualize the relationship. All analyses adhered to standard statistical protocols, and results were interpreted within the limitations of this pilot study.

### Statistical analysis (Prism 10)

To assess group-level differences in pulmonary decline, separate linear regression models were constructed for FGFR4 mutation-positive and mutation-negative patients in GraphPad Prism 10. The “Comparison of Fits” module was used to test whether a model with separate slopes provided a better fit than a shared-slope model. This comparison was performed using an F-test, with a significance threshold of *p* < 0.05. To quantify group differences in mean FEV₁% slope values, an unpaired t-test with Welch’s correction was applied. Standard deviations and 95% confidence intervals were reported.

### Spatial transcriptomic analysis of LAM lung tissue

To assess spatial *FGFR4* expression in LAM, we performed spatial transcriptomic profiling on FFPE lung tissue sections from two LAM patients (LAM1 and LAM2) from our previously published dataset (8). The spatialomics datasets, including raw sequencing data and processed files, have been deposited at NCBI GEO under the series reference GSE234885 (datasets GSM7476184 and GSM7476185). The original code is available in the GitHub repository (https://github.com/gautam-lk/RyanLab_LAM; commit ID eda3311). Briefly, sections (5 μm) were processed using the 10x Genomics Visium platform. Following H&E staining and imaging, libraries were prepared according to the manufacturer’s protocol and sequenced on an Illumina NovaSeq 6,000. Data processing was carried out with Space Ranger (v1.3.0), and unbiased clustering was performed in Seurat (v4.0). To ensure data quality and analytic consistency, standard quality control filters were applied. Only spatial transcriptomic spots with unique molecular identifier (UMI) counts between 500 and 15,000, and gene feature counts between 300 and 6,000, were retained for analysis. Cluster annotation was guided by the Human Lung Cell Atlas version 2 (HLCA v2) reference, part of the Human BioMolecular Atlas Program (HuBMAP), an NIH-funded initiative aimed at mapping the human body at single-cell resolution, using the Azimuth platform with filtered gene count matrices (14). *FGFR4* expression was spatially mapped and analyzed across annotated clusters, including fibroblast-rich regions, alveolar epithelial (AT2) cells, and vascular compartments, to contextualize its potential role in LAM pathogenesis.

### Cell-type enrichment analysis using Enrichr for cross-referencing

To cross-reference our cell type- annotation we have used the top differentially expressed genes (DEGs) in spatial transcriptomic cluster 3 from LAM1 and Cluster 4 from LAM2 expressing *FGFR4* and we conducted a complementary cell-type enrichment analysis using the Enrichr platform.^1^ The top 50 statistically significant DEGs from each cluster were selected based on adjusted *p*-values (adj. *p* < 0.05). These gene lists were analyzed against a broad panel of curated cell-type and tissue-specific reference libraries within Enrichr, encompassing human transcriptomic atlases and single-cell references.

## Results

Drawing on advances in liquid biopsy technology from the lung cancer field, we aimed to investigate whether LAM patients harbour detectable systemic genetic variants in circulating cells that could be associated with disease progression. PBMCs were isolated from seven LAM patients and NGS targeted mutation analysis performed using the Oncomine Comprehensive Assay v3 available from Thermo Fisher Scientific. This high-throughput, NGS panel is widely standardized for clinical application to evaluate potential variations in over 160 cancer-related genes, including key oncogenes, tumour suppressor genes, and genes associated with tumorigenesis pathways. This highly multiplexed reference includes 74 variants across 62 genes relevant to solid tumors, comprising 37 SNVs, 4 INDELs, 18 deletions, 5 insertions, and 10 translocations. Notably, 65 of these variants represent FDA-approved drug targets, ensuring comprehensive and clinically relevant benchmarking. FGFR4 was prioritized for further analysis based on its known biological relevance and its spatial expression pattern in LAM tissue.

**Figure 1:**
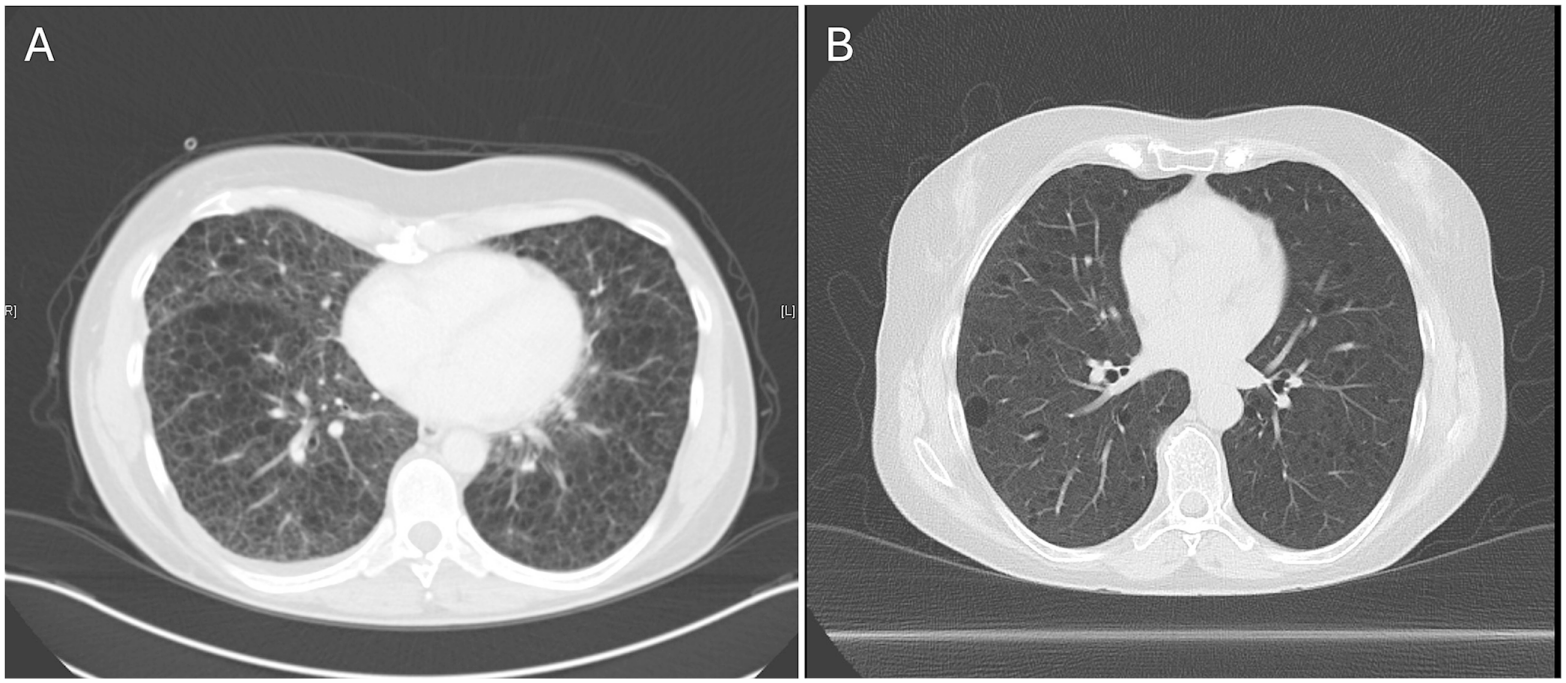
Representative lung CT-scans of STLAM1 and STLAM7. **(A)** CT images from STLAM1, with a high allelic frequency (99%) of the FGFR4 p.Gly388Arg polymorphism, demonstrate advanced cystic destruction. **(B)** In contrast, STLAM7, with no FGFR4 p.Gly388Arg polymorphism, exhibits bilateral cysts of varying sizes alongside preserved lung areas.

The clinical and genetic characteristics of the study cohort are summarized in Table 1. To our knowledge, this is the first report describing FGFR4 polymorphisms in PBMCs from patients with LAM, suggesting potential systemic involvement. The FGFR4 p.Gly388Arg polymorphism (chr5:176520243) was identified in 4 of 7 patients, with variant allele frequencies of 49, 49, 51, and 99%. The highest frequency (99%) was observed in patient STLAM1, who had extensive bullous lung disease (Figure 1), a history of pneumothorax, and lymphangioleiomyoma, and was undergoing treatment with sirolimus. STLAM2 also carried the FGFR4 variant at 49% allelic frequency and presented with cystic lung changes, pleural effusions, and renal angiomyolipomas (AML), also receiving sirolimus. STLAM6 exhibited similar systemic involvement, including pleural effusions, chylothorax, and abdominal lymphadenopathy, with a 49% FGFR4 variant frequency, but no detectable TSC2 mutation. Patient STLAM3 had a 51% frequency of the FGFR4 variant and demonstrated cystic lung changes but was not receiving treatment.

**Table 1:** Genetic characteristics of LAM patients.

Patient ID	STLAM1	STLAM2	STLAM3	STLAM4	STLAM5	STLAM6	STLAM7
Age range	50–55	56–60	56–60	65–70	50–55	56–60	65–70
Gender	Female	Female	Female	Female	Female	Female	Female
LAM type	Sporadic	Sporadic	Sporadic	Sporadic	Sporadic	Sporadic	Sporadic
FGFR4 mutation	Yes	Yes	Yes	No	No	Yes	No
FGFR4 allelic frequency (%)	99	49	51	N/A	N/A	49	N/A
FGFR4 mutation chromosome locus	chr5:176520243	chr5:176520243	chr5:176520243	N/A	N/A	chr5:176520243	N/A
FGFR4 mutation type	SNP	SNP	SNP	N/A	N/A	SNP	N/A
Mutation coverage	1989,1992, 1959, 1995	1989, 1992, 1959, 1995	1989, 1992, 1959, 1995	N/A	N/A	1989, 1992, 1959, 1995	N/A
TSC2 mutation	No	Yes	No	No	No	No	No
TSC2 allelic frequency (%)	N/A	56	N/A	N/A	N/A	N/A	N/A
TSC2 mutation chromosome locus	N/A	chr16:2138268	N/A	N/A	N/A	N/A	N/A
TSC2 mutation type	N/A	INDEL	N/A	N/A	N/A	N/A	N/A
TSC2 mutation coverage	N/A	1993	N/A	N/A	N/A	N/A	N/A
Clinical features	Severe bilateral bullous lung disease; history of 1 pneumothorax; known lymphangioleiomyoma; on LTOT, prior pregnancy (1)	Cystic lung remodeling; history of pleural effusions; history of AML; on LTOT, Hx of adnexectomy	Cystic lung remodeling; history of pneumothorax and ovarian cysts; prior pregnancy (1)	Cystic lung remodeling; history of AML; on LTOT	Multiple small lung cysts with bilateral ground-glass opacities and infiltrative changes; history of chylous pleural effusions and AML; on LTOT	Thin-walled central lung cysts with ground-glass infiltrates; Hx of chylous pleural effusions and ascites; Hx of abdominal lymphadenopathy; Hx of suspected liver hemangiomas; Hx of adnexectomy, prior pregnancy (1)	Multiple lung cysts; Hx of 1 pneumothorax; Hx of chylous pleural effusions and ascites; known lymphangioleiomyoma; history of adnexectomy
Treatment	Sirolimus	Sirolimus	None	None	Sirolimus	Sirolimus	None
Sirolimus start (years from diagnosis)	Year 7	Year 0–2, Reinitiation Year 4			Year 0	Year 1	
LTOT start (years from diagnosis)	Year 3	Year 3		Year 0	Year 0		

*AML = Angiomyolipoma; FGFR4 = Fibroblast Growth Factor Receptor 4; Hx = History of; INDEL = Insertion/Deletion; LTOT = Long-Term Oxygen Therapy; N/A = Not Applicable; SNP = Single Nucleotide Polymorphism; TSC2 = Tuberous Sclerosis Complex.

Additional genetic findings included a *TSC2* mutation (p.Ile1735HisfsTer40; chr16:2138268) in STLAM2 with a 56% allelic frequency, and a *BRCA2* variant (p.Ile2177MetfsTer13; chr13:32915019) in STLAM1 at low frequency (4%). STLAM2 also harbored a *NOTCH3* variant (p.Asp1086Ala) at a low allelic frequency (6.83%). Patients STLAM4, STLAM5, and STLAM7 did not carry the *FGFR4* variant but exhibited cystic lung disease in the absence of detectable systemic TSC2 mutations (Supplementary Table 1).

**Figure 2:**
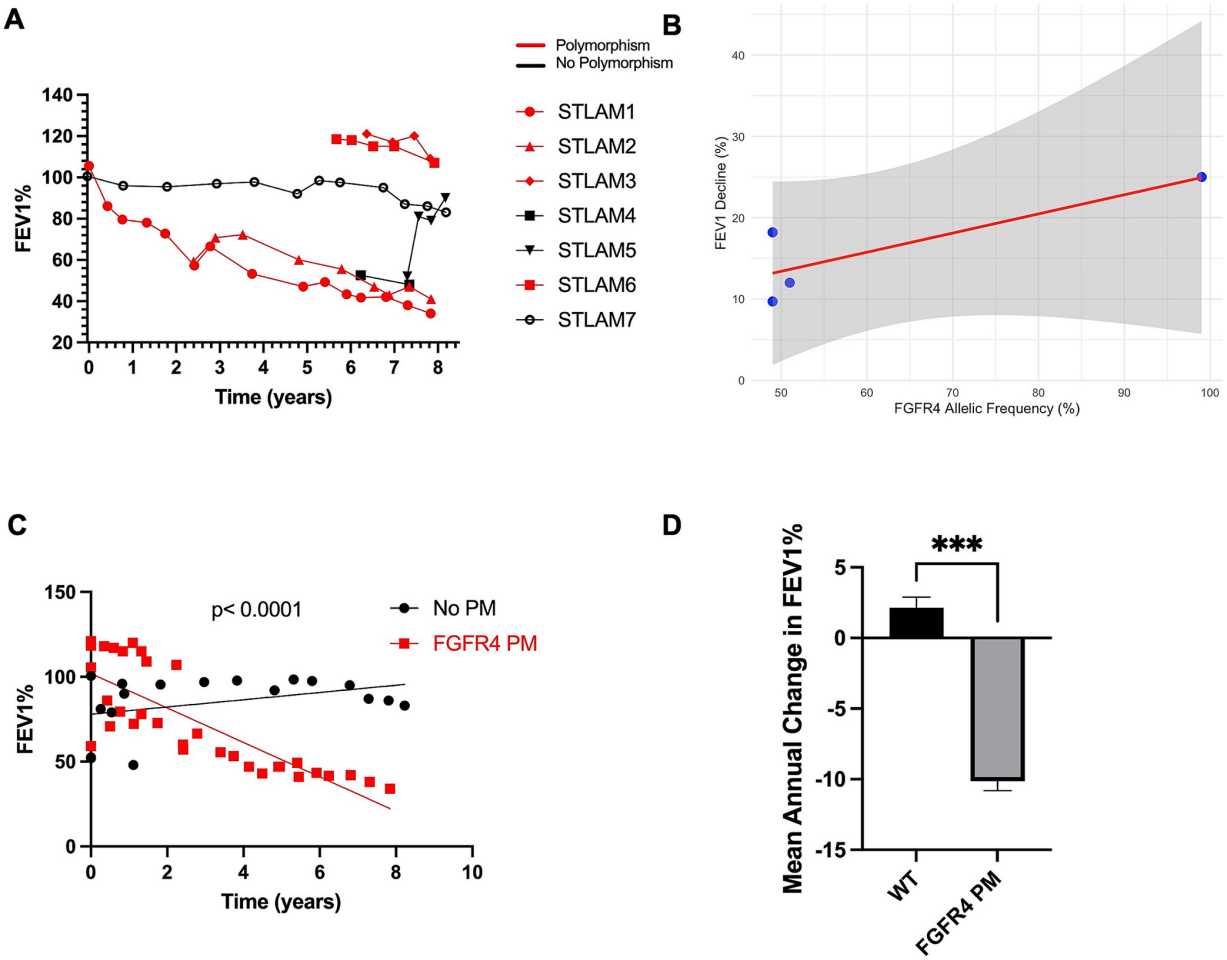
Longitudinal lung function decline in LAM patients stratified by FGFR4 mutation status. **(A)** Individual patient trajectories of FEV₁% over time for seven sporadic-LAM patients, stratified by FGFR4 p.Gly388Arg polymorphism status. Patients harboring the variant generally show steeper declines. **(B)** The scatter plot shows the relationship between FGFR4 allelic frequency (%) and lung function decline (FEV1%) among LAM patients carrying the FGFR4 polymorphism. Each point corresponds to an individual patient, with the red regression line highlighting the observed trend. A strong positive correlation (r = 0.847, *p* = 0.153) was observed, though it did not reach statistical significance. These preliminary findings suggest that higher FGFR4 allelic frequencies may be associated with accelerated lung function decline. The analysis was performed using Pearson’s correlation and a linear regression model. **(C)** Comparison of linear regression slopes between wild-type (WT) and FGFR4 polymorphism (PM) groups. Individual regressions showed a steep negative slope in the PM group (−10.15% per year; 95% CI: −12.83 to −7.48; R^2^ = 0.66), and a mildly positive slope in the WT group (+2.14% per year; 95% CI: −0.62 to 4.89; R^2^ = 0.14). Global slope comparison using GraphPad Prism’s “Comparison of Fits” yielded a statistically significant difference (F(1,47) = 42.48, *p* < 0.0001), confirming that patients with the FGFR4 p.Gly388Arg polymorphism exhibit a significantly steeper decline in FEV₁% over time. **(D)** Bar chart comparing the mean annual FEV₁% change between wild-type (WT) and FGFR4 polymorphism-positive (PM) patients. WT patients (n = 3) showed a modest increase in lung function over time (mean = +2.14%), whereas FGFR4 PM patients (*n* = 4) experienced a significant decline (mean = −10.15%). The difference between the groups was statistically significant (*p* = 0.0001, unpaired t-test with Welch’s correction; t = 12.34, df = 4.48), with a 95% confidence interval for the difference ranging from −14.94 to −9.63. Error bars represent standard error of the mean (SEM).

To further investigate the relationship between *FGFR4* polymorphisms and disease progression, we determined the correlation between the allelic frequency of the FGFR4 p.Gly388Arg polymorphism and the rate of decline in lung function (FEV1%) over time. In total, we analyzed 52 PFTs from 7 patients, with each patient contributing between 2 and 10 longitudinal measurements. Figure 2A highlights the longitudinal changes in FEV1% for each patient. Pearson’s correlation analysis was performed to examine the association between FGFR4 allelic frequency and the rate of lung function decline. Our results revealed a strong positive correlation (r = 0.85), though the relationship did not reach statistical significance (*p* = 0.15) (Figure 2B). This finding suggests that higher allelic frequency of the FGFR4 variant may be associated with a faster decline in lung function in LAM patients. Additionally, we analyzed the relationship between *FGFR4* variant status (presence vs. absence) and FEV1% decline. This analysis revealed a moderate positive correlation (r = 0.55, *p* = 0.20) between mutation status and lung function decline, suggesting that patients harboring *FGFR4* mutations may experience a more pronounced decline in FEV1% compared to those without mutations (Supplementary Figure 1). Although the results do not quite reach statistical significance due to the sample size, limited by access to sufficient patients with this rare disease, the observed trends highlight the potential importance of *FGFR4* mutations in disease progression.

Further linear regression analysis demonstrated a pronounced negative slope in the FGFR4 mutation-positive group (slope = −10.15; 95% CI: −12.83 to −7.48; R^2^ = 0.66), suggesting a consistent annual decline in lung function. In contrast, the mutation-negative group showed a slight positive slope (slope = +2.14; 95% CI: −0.62 to 4.89; R^2^ = 0.14), which may indicate relative stability or marginal improvement over time. A comparison of the two models confirmed that the difference in slopes was statistically significant (*F* (1,47) = 42.48, *p* < 0.0001) (Figure 2C). Complementing this, an unpaired t-test with Welch’s correction comparing group means revealed a significant difference (t (4.48) = 12.34, *p* = 0.0001), with a mean difference of −12.29 (95% CI: −14.94 to −9.634) (Figure 2D).

**Figure 3:**
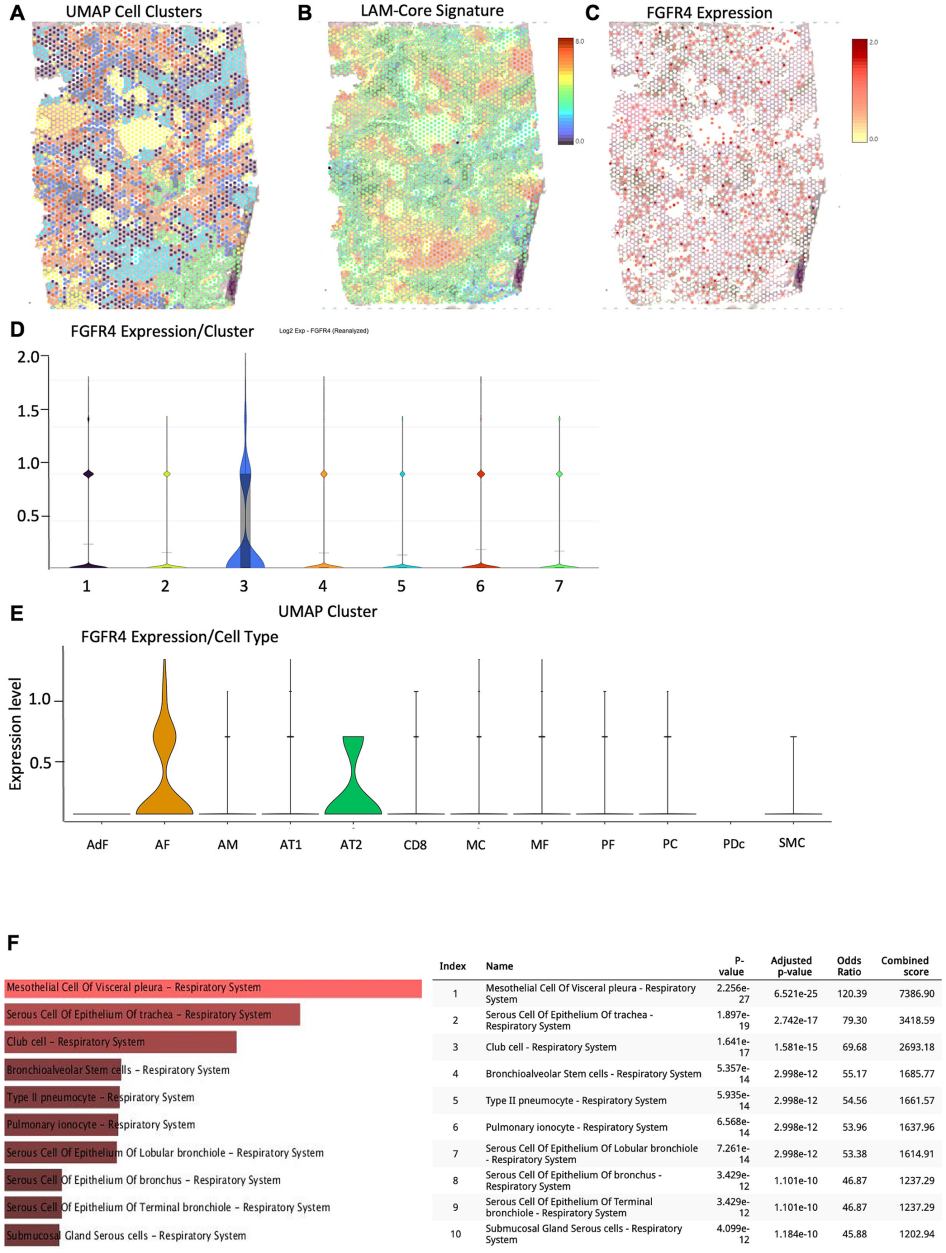
Spatial transcriptomic mapping of FGFR4 expression in LAM Donor 1. **(A)** Unbiased UMAP clustering of spatial transcriptomics data from FFPE lung tissue (LAM1) revealed distinct molecular regions (8). **(B)** Projection of a previously published LAM-Core transcriptional signature (e.g., PMEL, ACTA2, ESR1, VEGFD) highlights its spatial distribution across tissue. **(C)** Spatial projection of FGFR4 expression shows enriched signal in discrete regions. **(D)** Violin plot of FGFR4 expression across UMAP-defined clusters, identifying Cluster 3 as the primary FGFR4-expressing compartment. **(E)** Azimuth-based annotation using HuBMAP HCLA v2 reference. Violin plot of FGFR4 expression by annotated cell type, showing dominant expression in alveolar fibroblasts (AF) and alveolar type 2 (AT2) epithelial cells. **(F)** Cell-type enrichment analysis using Enrichr cross-referencing confirmed overlap of FGFR4-associated gene signatures with pulmonary epithelial and stromal populations, including visceral mesothelial cells, club cells, AT2 pneumocytes, and bronchiolar epithelial cells, further supporting the epithelial-stromal localization of FGFR4 expression in LAM. Abbreviations: AdF, adventitial fibroblasts; AF, alveolar fibroblasts; AM, alveolar macrophages; AT1, alveolar type 1 epithelial cells; AT2, alveolar type 2 epithelial cells; CD8, CD8^+^ T cells; MC, mast cells; MF, myofibroblasts; PF, pericyte-like fibroblasts; PC, pericytes; PDc, plasmacytoid dendritic cells; SMC, smooth muscle cells.

To contextualize the spatial relevance of *FGFR4* expression in LAM pathophysiology, we re-analyzed our spatial transcriptomic data from FFPE lung tissue of two sporadic LAM donors (LAM1 and LAM2) from our previously published dataset (GEO: GSE234885) (8). Unbiased clustering identified molecularly distinct compartments across both tissue samples. In LAM1, *FGFR4* expression was primarily localized to Cluster 3, which corresponded to alveolar fibroblasts (AF) and alveolar type II (AT2) epithelial cells, as defined by Azimuth-based annotation using the Human Lung Cell Atlas v2 (HLCA v2) reference (Figures 3A–E). In LAM2, *FGFR4* expression was localized in Cluster 4 and was consistent with the findings in LAM1. Azimuth-based annotation in LAM2 revealed *FGFR4* expression across alveolar fibroblasts, AT2 epithelial cells, and endothelial cells (Supplementary Figure 2E). Taken together, these two cell types are recognized contributors to LAM pathogenesis via their roles in tissue remodeling, fibrogenesis, and epithelial–stromal crosstalk. *FGFR4* was notably absent from the LAM-core cluster (8), which was demarcated by expression of ACTA2, PMEL, VEGFD, and other canonical LAM markers like previously published (8, 15). Spatial projection of *FGFR4* expression, independent of clustering, confirmed enrichment in fibroblast- and epithelial-rich areas (Figure 3C). Cluster 3 also showed statistically significant enrichment of differentially expressed genes (adjusted *p* < 0.05), supporting its biological distinctiveness and relevance to disease remodeling mechanisms (Supplementary Data Sheet 2). To further substantiate the cell-type identity of *FGFR4*-enriched clusters, we performed cross-referencing of the top differentially expressed genes (DEGs) from Cluster 3 using (Figure 3F) Enrichr-based cell-type enrichment analysis. This analysis drew on multiple human and murine transcriptomic and proteomic reference libraries, including single-cell atlases and tissue-specific annotations. The resulting enrichment profile demonstrated strong signatures of respiratory epithelial cell types—including mesothelial cells, club cells, bronchioalveolar stem cells, and type II pneumocytes—providing validation of Azimuth and HLCA v2-based annotations. Consistent with the findings in LAM1, Enrichr-based cell-type enrichment analysis of DEGs in Cluster 4 of donor LAM 2 showed also strong signatures of respiratory epithelial cell types (Supplementary Figure 2F; Supplementary Data Sheet 3). To further validate these findings, we cross-referenced *FGFR4* expression patterns with data from the LAM Cell Atlas single-cell transcriptomic map^2^. This external dataset confirmed *FGFR4* expression in alveolar fibroblasts and alveolar type II (AT2) epithelial cells, consistent with our spatial transcriptomics results (Supplementary Figure 3). This orthogonal confirmation strengthens the interpretation that *FGFR4* is preferentially expressed in stromal and epithelial compartments relevant to LAM pathology. This spatial localization provides a functional map for where the *FGFR4* p.Gly388Arg gain-of-function polymorphism may exert its effects (16–23).

## Discussion

By evaluating PBMC, this study highlights a potential systemic involvement in LAM progression and supports the need to further examine LAM as a systemic neoplastic-like condition. Previous studies have established TSC2 mutations as the primary driver of LAM. However, our preliminary findings suggest that the FGFR4 p.Gly388Arg gain-of-function polymorphism in PBMCs may reflect either germline origin or modifications in the immune cell repertoire and therefore may act as potential co-modifiers of disease, warranting further investigation into their role beyond the mTOR pathway. This aligns with research indicating that FGFR4 polymorphisms, particularly the FGFR4 p.Gly388Arg variant, negatively impact the prognosis of neoplastic diseases. Importantly, spatial transcriptomic analysis revealed that FGFR4 expression localizes to alveolar fibroblasts and AT2 epithelial cells—key stromal and epithelial compartments implicated in LAM pathophysiology—providing a tissue-level map for where this gain-of-function polymorphism may exert its pathogenic effects. A systematic review by Kim et al. demonstrated that FGFR4 p.Gly388Arg polymorphisms are associated with worse outcomes in cancer patients (21). Although LAM is typically a slow-progressing neoplasm, the discovery of this polymorphism in immune cells in peripheral blood may have several important implications.

It is important to note that while the FGFR4 p.Gly388Arg variant has been identified at lower allelic frequencies in population studies, including a large cohort of 16,179 controls, it is present in approximately 33% of the global population, with variability depending on ethnicity (19, 24). Data from the Genome Aggregation Database (gnomAD) further show that the allele frequency reaches 30.5% in European (non-Finnish) females and 26.8% overall (Supplementary Figure 5). This is an important fact, but its high population prevalence does not imply benignity. Many carriers may remain asymptomatic, and the development of disease may require additional somatic events or second-hit mutations that converge on downstream pathways modulated by this polymorphism. Notably, this variant is a functional polymorphism that alters the receptor’s transmembrane domain, exposing a STAT3 binding site and enhancing downstream signaling, including the PI3K/AKT/mTOR pathway (25). Our small cohort of LAM patients exhibited a higher observed frequency of this variant. However, due to the limited sample size, these findings are exploratory and lack the statistical power for definitive conclusions. Accordingly, they should not be interpreted as evidence of enrichment but instead warrant further investigation in larger cohorts to assess their clinical relevance. Despite the small cohort the FGFR4 p.Gly388Arg variant was detected in 4 out of 7 patients (56%). This polymorphism has been repeatedly implicated in promoting cell motility and lymph node metastasis in several malignancies, including lung, breast, head and neck, gastric, and colorectal cancers (17–19, 21–23, 26, 27). A metaanalysis including 8,555 subjects demonstrated an increased susceptibility to cancer in carriers of this variant (28), while two additional meta-analyses associated it with poor prognosis in cancer patients (21, 25). Functional studies have shown that the p.Gly388Arg substitution activates downstream signaling pathways. Specifically, it promotes STAT pathway activation, contributing to epithelial-to-mesenchymal transition (20, 22). The substitution of glycine with arginine at codon 388 alters the structure of the FGFR4 receptor and has been shown in *in vivo*, *in vitro*, and *in silico* models to induce MAPK activation, which may drive increased cell proliferation, survival, and tumorigenesis (17, 27).

Given LAM’s neoplastic-like characteristics, it is plausible that a subset of LAM patients carrying this FGFR4 variant may experience more severe disease progression. For example, patient STLAM1, who exhibited a 99% allelic frequency for this mutation, had a more aggressive clinical course, including the development of bullous cysts necessitating lung transplantation. In contrast, patient STLAM7, who lacked the FGFR4 variant, experienced a milder disease trajectory during a comparable follow-up period. This contrast underscores the potential of FGFR4 p.Gly388Arg to exacerbate disease severity and accelerate pulmonary decline, though further validation is required. Importantly, FGFR4 p.Gly388Arg may interact with established LAM-related pathways. Crosstalk between FGFR4 and mTOR signaling is a compelling area for future research. FGFR4 activation could potentiate the effects of TSC2 mutations by engaging downstream pathways such as MAPK/ERK and PI3K/AKT, both of which are known to regulate mTOR signaling. This interaction may contribute to unchecked cellular proliferation and survival, facilitating disease progression in LAM. These insights support the hypothesis that FGFR4, alongside mTOR, could represent a future therapeutic target, pending further mechanistic and clinical validation.

Detection of FGFR4 mutations in PBMCs, including T cells, B cells, and natural killer (NK) cells, suggests a possible role for immune dysregulation in LAM. While the role of FGFR4 mutations in immune cells in LAM remains unclear, research in other neoplastic conditions suggests that such mutations can contribute to disease progression. Although five of the seven patients in our study underwent lung biopsies as part of their clinical evaluation, archived tissue samples were not viable for DNA extraction, precluding parallel sequencing of affected lung tissue. We acknowledge this as a key limitation of our study. Future studies should prioritize matched analysis of tissue and blood to assess for somatic mosaicism, clonal expansion, or tissue-specific mutational patterns that may underlie clinical heterogeneity in LAM. Notably, patient STLAM2 harbored both a TSC2 mutation and the FGFR4 variant but did not present with classic features of TSC, suggesting a complex interplay between these genetic alterations in LAM. The presence of the TSC2 mutation in PBMCs also raises the possibility of systemic involvement, potentially acting alongside FGFR4 variants to amplify pro-fibrotic signaling or support abnormal proliferation of LAM cells.

Together, these findings introduce FGFR4 as a potential genetic co-modifier in LAM pathogenesis. The data suggest a role for FGFR4 in contributing to disease heterogeneity and progression in a subset of patients. These preliminary observations highlight the need for larger cohort studies and mechanistic *in vitro* and *in vivo* investigations to validate our findings and explore the functional implications of FGFR4 and mTOR signaling interactions in LAM. In conclusion, this study provides early evidence supporting a role for FGFR4 in LAM and sets the foundation for hypothesis-driven research to further elucidate its impact on disease progression and potential as a therapeutic target.

## Data Availability

The spatial transcriptomics datasets generated and analyzed in this study, including raw sequencing data and processed files, have been deposited in the NCBI Gene Expression Omnibus (GEO) under accession number GSE234885 (GSM7476184 and GSM7476185). Targeted sequencing data of peripheral blood mononuclear cells (PBMCs) from seven sporadic LAM patients, including BAM and BAI files aligned to the GRCh37 (hg19) reference genome, allelic frequency tables, and associated clinical metadata, are publicly available in the KocGünel Lab GitHub repository: https://github.com/skocguen/KocG-nelLAB_FGFR4LAM. Analysis code used for spatial transcriptomic processing and visualization is available at: https://github.com/gautam-lk/RyanLab_LAM (commit ID: eda3311).

## Footnotes

^1^ https://maayanlab.cloud/Enrichr/

^2^(https://research.cchmc.org/pbge/lunggens/LCA/lam_t_genequery.html?tissue=lung&AID=LCAANALYSIS00000005&tab=gene&ensid=ENSG00000160867&geneid=FGFR4)
