# Supplementary material for: FGFR4 p.Gly388Arg polymorphism in PBMCs of LAM patients: findings of a pilot study
Source: Front Med (Lausanne). 2025 Jul 24;12:1544910. doi: 10.3389/fmed.2025.1544910 (PMC12328168; doi:10.3389/fmed.2025.1544910)
Supplement: Supplementary file 2 [file Data_Sheet_1.PDF]

Supplementary Figures

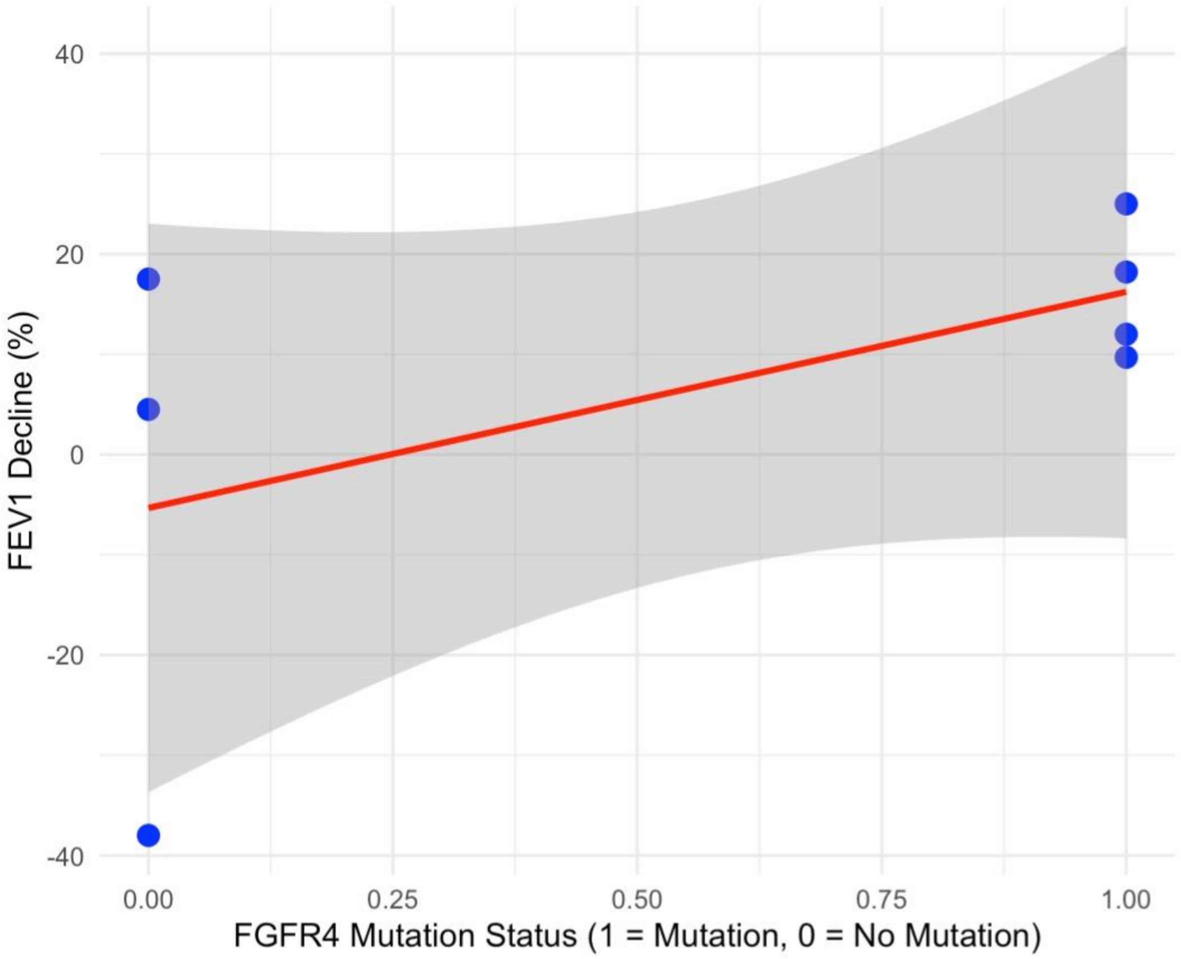

Supplemental Figure 1: Correlation between FGFR4 polymorphism status and FEV1% decline in LAM patients. The scatter plot illustrates the relationship between FGFR4 polymorphism status (1 = polymorphism present, 0 = polymorphism absent) and the percentage decline in lung function (FEV1%) among LAM patients. Each point represents an individual patient, and the red regression line indicates the trend. A moderate positive correlation ($r = 0.551$, $p = 0.200$) was observed, although this did not reach statistical significance. These findings suggest that patients with the FGFR4 polymorphism may experience a greater decline in lung function compared to those without. Data points were analyzed using Pearson's correlation and a linear regression model.

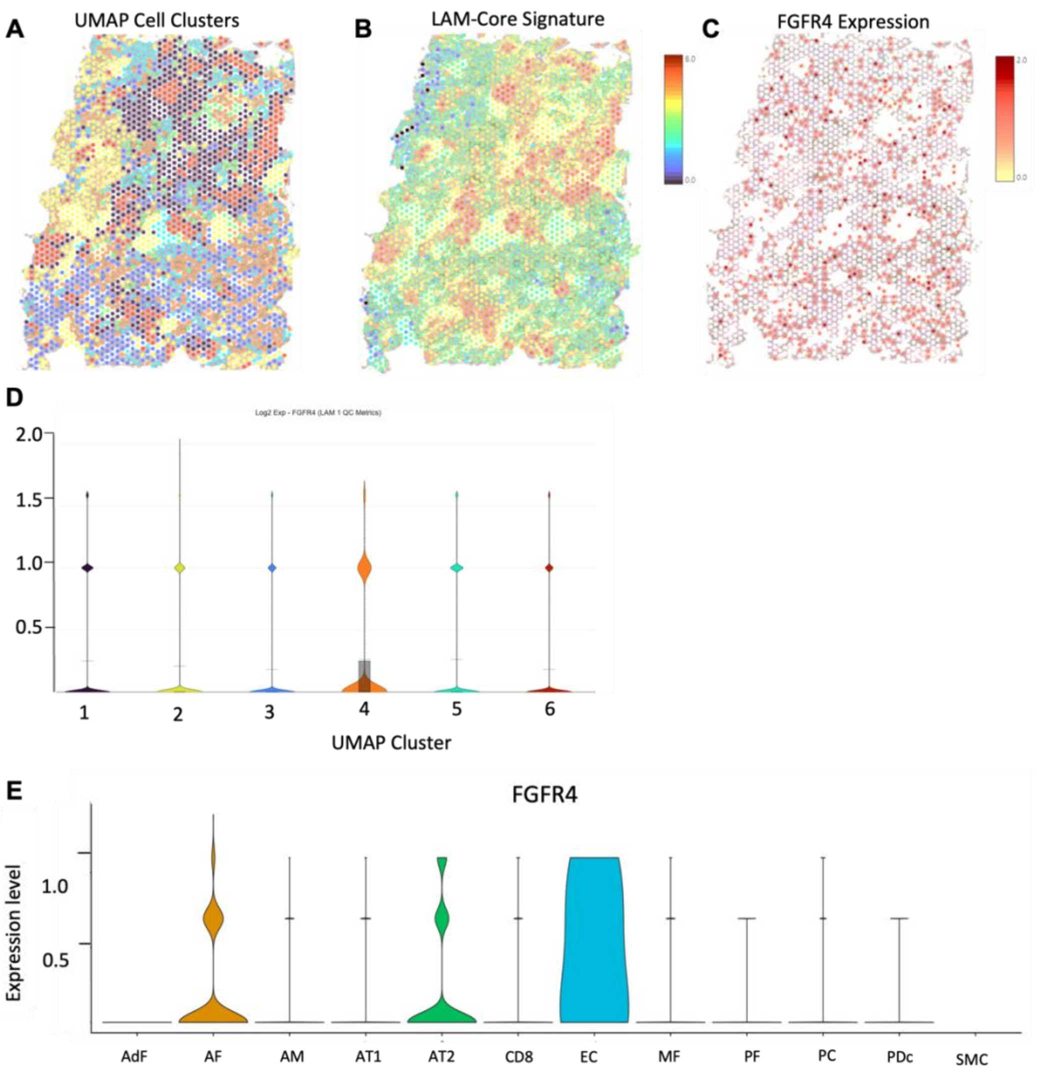

F

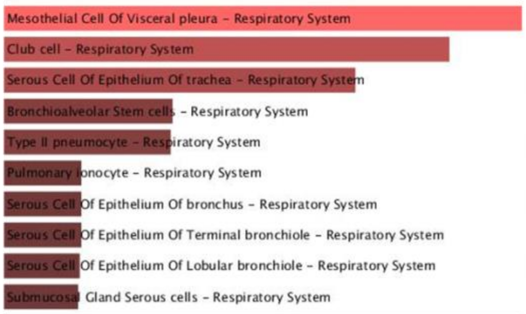

Index	Name	P-value	Adjusted p-value	Odds Ratio	Combined score
1	Mesothelial Cell Of Visceral pleura - Respiratory System	4.181e-26	8.655e-24	171.36	10013.82
2	Club cell - Respiratory System	1.201e-19	1.243e-17	115.22	5019.58
3	Serous Cell Of Epithelium Of trachea - Respiratory System	1.173e-17	8.092e-16	101.22	3945.84
4	Bronchioalveolar Stem cells - Respiratory System	8.567e-14	3.889e-12	75.60	2274.63
5	Type II pneumocyte - Respiratory System	9.394e-14	3.889e-12	74.77	2242.90
6	Pulmonary ionocyte - Respiratory System	7.268e-12	1.763e-10	62.64	1606.44
7	Serous Cell Of Epithelium Of bronchus - Respiratory System	7.268e-12	1.763e-10	62.64	1606.44
8	Serous Cell Of Epithelium Of Terminal bronchiole - Respiratory System	7.268e-12	1.763e-10	62.64	1606.44
9	Serous Cell Of Epithelium Of Lobular bronchiole - Respiratory System	7.870e-12	1.763e-10	61.97	1584.51
10	Submucosal Gland Serous cells - Respiratory System	8.516e-12	1.763e-10	61.32	1563.09

Supplemental Figure 2: Spatial transcriptomic mapping of FGFR4 expression in LAM Donor 2. A) UMAP-based clustering of spatial transcriptomic data from FFPE lung tissue of LAM Donor 2 (LAM2), showing molecularly distinct regions across the tissue section. B) Projection of the previously defined LAM-Core gene signature (including PMEL, ACTA2, ESR1, and VEGFD) reveals LAM-enriched domains. C) Spatial map of FGFR4 expression shows patchy but regionally enriched signal across the tissue section. D) Violin plot of FGFR4 expression across UMAP-defined clusters 1–6, with the highest expression observed in Cluster 4. E) Violin plot showing FGFR4 expression by cell type, annotated via Azimuth-based

mapping using the Human Lung Cell Atlas v2 (HuBMAP HCLA v2) reference, indicating prominent expression in alveolar fibroblasts (AF), alveolar type 2 epithelial cells (AT2), and capillary endothelial cells (EC). F) Cell-type enrichment analysis using Enrichr cross-referencing confirmed overlap of FGFR4-associated gene signatures with pulmonary epithelial and stromal populations, including visceral mesothelial cells, club cells, AT2 pneumocytes, and bronchiolar epithelial cells, further supporting the epithelial-stromal localization of FGFR4 expression in LAM. Abbreviations: AdF, adventitial fibroblasts; AF, alveolar fibroblasts; AM, alveolar macrophages; AT1, alveolar type 1 epithelial cells; AT2, alveolar type 2 epithelial cells; CD8, CD8$^+$ T cells; EC, capillary endothelial cells; MF, myofibroblasts; PF, pericyte-like fibroblasts; PC, pericytes; PDc, plasmacytoid dendritic cells; SMC, smooth muscle cells.

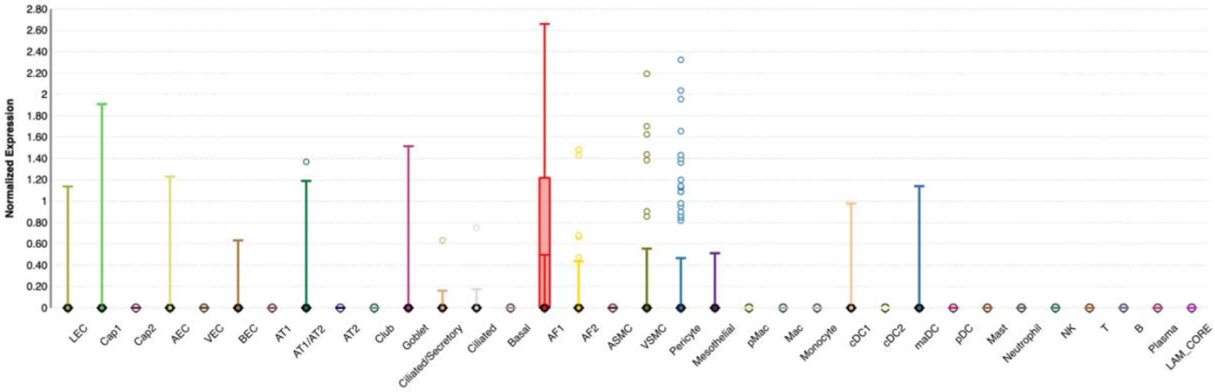

Supplemental Figure 3: FGFR4 expression across lung cell populations in the LAM Cell Atlas. Box plot showing normalized FGFR4 expression across annotated lung cell types from the LAM Cell Atlas. Notable expression was observed in alveolar fibroblast populations (AF1, AF2), alveolar type 1 (AT1) and type 2 (AT2) epithelial cells, and endothelial subtypes (Cap1, AEC). Expression in LAM_CORE cells was minimal. This distribution supports the relevance of FGFR4 in stromal and epithelial niches implicated in LAM pathophysiology.

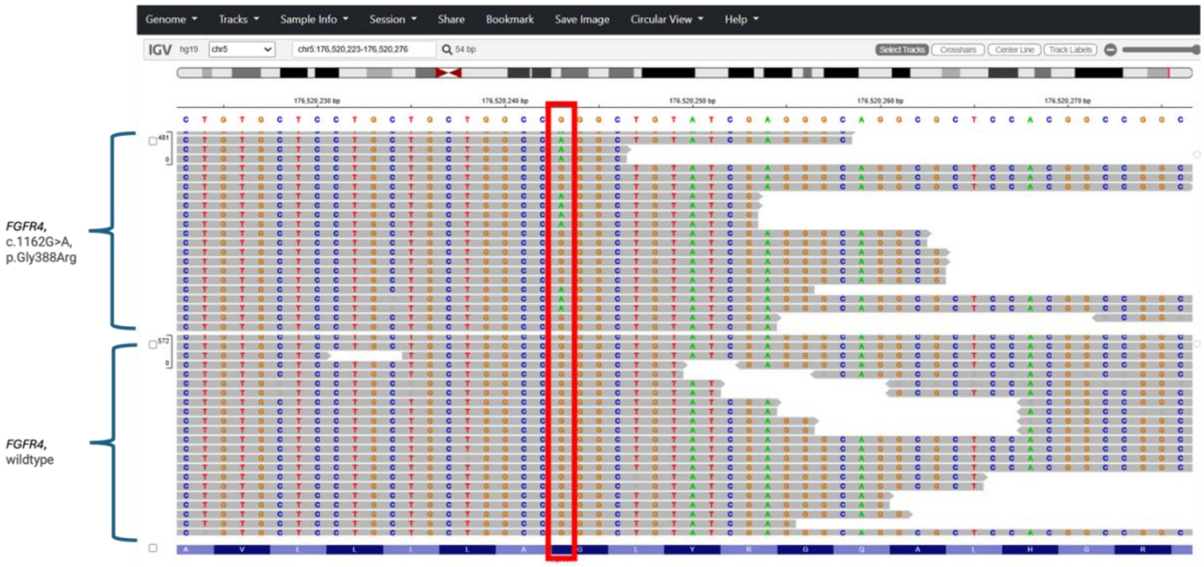

Supplemental Figure 4: FGFR4 expression across lung cell populations in the LAM Cell Atlas. Integrated Genomics Viewer (IGV) showing aligned sequencing reads at chromosome 5 (chr5:176520243, hg19), highlighting the c.1162G>A (p.Gly388Arg) variant in the FGFR4 gene. The variant is clearly visible as a G>A substitution (boxed in red) in sequencing reads from a mutation-positive patient (top panel), in contrast to the reference G base observed in a wildtype control sample (bottom panel). Each row represents an individual sequencing read, and bases are color-coded by nucleotide identity. The altered amino acid position (glycine to arginine) is shown in the bottom sequence track, confirming the coding impact of the variant. This figure supports the variant detection reported in PBMC-derived DNA via NGS analysis.

Genetic Ancestry Group Frequencies

gnomAD | HGDP | 1KG | Local Ancestry

Genetic Ancestry Group		Allele Count	Allele Number	Number of Homozygotes	Allele Frequency
East Asian		2305	5134	508	0.4490
South Asian		1765	4818	332	0.3663
Admixed American		5046	15284	891	0.3301
European (Finnish)		3441	10576	571	0.3254
Ashkenazi Jewish		1113	3470	189	0.3207
	Overall	20708	67940	3207	0.3048
European (non-Finnish)	XX	12009	39310	1836	0.3055
	XY	8699	28630	1371	0.3038
Amish		248	912	32	0.2719
Remaining		574	2112	74	0.2718
Middle Eastern		66	294	4	0.2245
African/African American		5519	41514	399	0.1329
XX		20569	77750	3057	0.2646
XY		20216	74304	3150	0.2721
**Total**		**40785**	**152054**	**6207**	**0.2682**

Supplemental Figure 5: Allele Frequency of FGFR4 p.Gly388Arg Variant Across Genetic Ancestry Groups in gnomAD. Summary table from gnomAD v4.1.0 (https://gnomad.broadinstitute.org/variant/5-177093242-G-A?dataset=gnomad_r4) showing allele count, total allele number, number of homozygotes, and allele frequency for the FGFR4 p.Gly388Arg (c.1162G>A) variant across multiple genetic ancestry groups. The overall allele frequency in the total population is 26.8% (0.2682), with the highest frequency observed in the East Asian population (44.9%). Among European (non-Finnish) individuals, the variant is found at a frequency of 30.5% in XX (female) individuals and 30.3% in XY (male) individuals. This contextualizes the observed variant prevalence in our LAM cohort relative to general population data.
